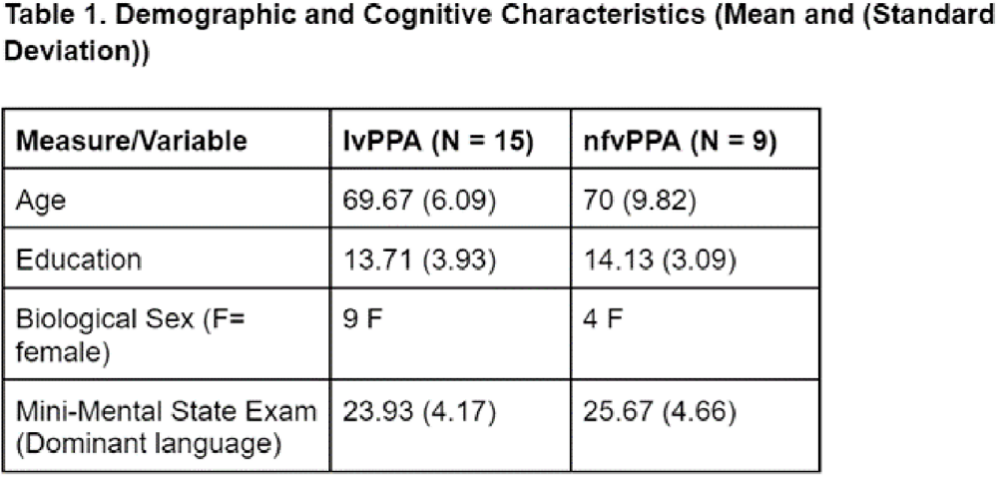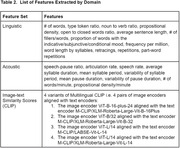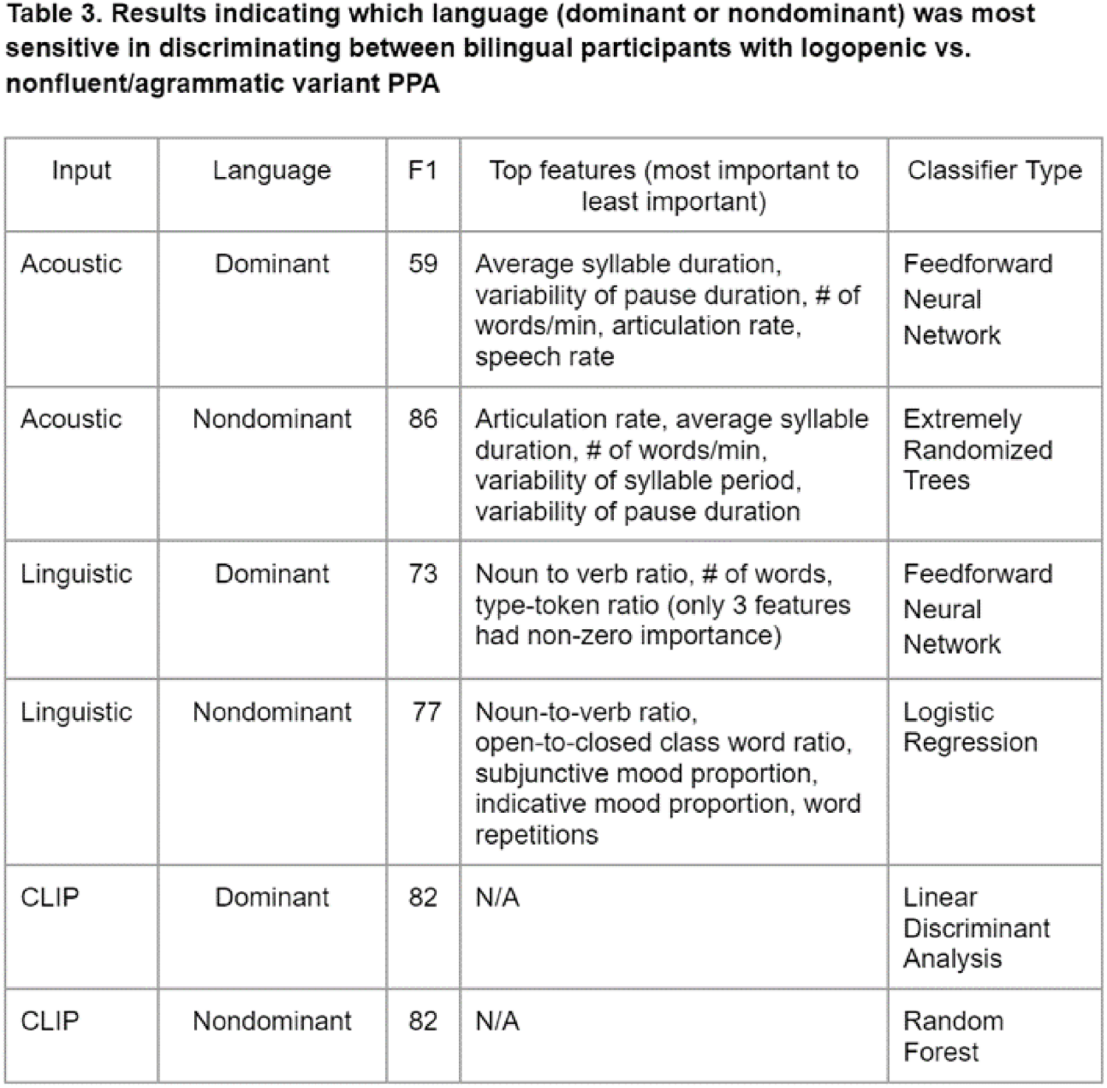# Can a picture description differentiate the nonfluent/agrammatic and logopenic variants of primary progressive aphasia?: Evidence from Catalan‐Spanish bilinguals

**DOI:** 10.1002/alz.094356

**Published:** 2025-01-09

**Authors:** Lokesha Pugalenthi, Núria Montagut, Sonia Karin Marques‐Kiderle, Camille Wagner Rodriguez, Ami Iyer, Jan Christian Holst Chaires, Whendy Avila Motta, Júlia Filella‐Merce, Nuole Zhu, Sara Rubio‐Guerra, Ignacio Illán‐Gala, Sergi Borrego‐Écija, Albert Lladó, Juan Fortea, Alberto Lleo, Raquel Sanchez‐Valle, Miguel A Santos‐Santos, Stephanie M Grasso

**Affiliations:** ^1^ Rice University, Houston, TX USA; ^2^ Alzheimer’s disease and other cognitive disorders Unit. Hospital Clínic. Fundació Clínic per a la Recerca Biomèdica, IDIBAPS, Universitat de Barcelona, Barcelona Spain; ^3^ Sant Pau Memory Unit, Hospital de la Santa Creu i Sant Pau ‐ Biomedical Research Institute Sant Pau ‐ Universitat Autònoma de Barcelona, Spain, Barcelona Spain; ^4^ University of Texas, Austin, TX USA; ^5^ Hospital de la Santa Creu i Sant Pau ‐ Biomedical Research Institute Sant Pau ‐ Autonomous University of Barcelona, Barcelona, Catalonia Spain; ^6^ Alzheimer’s disease and other cognitive disorders Unit. Hospital Clínic de Barcelona; FRCB‐IDIBAPS; University of Barcelona, Barcelona Spain; ^7^ Alzheimer’s disease and other cognitive disorders unit, Hospital Clínic, IDIBAPS, Barcelona Spain; ^8^ Sant Pau Memory Unit, Hospital de la Santa Creu i Sant Pau, Biomedical Research Institute Sant Pau, Universitat Autònoma de Barcelona, Barcelona Spain; ^9^ Alzheimer’s Disease and Other Cognitive Disorders Unit, Hospital Clínic, Institut d'Investigacions Biomediques August Pi i Sunyer (IDIBAPS), Barcelona Spain

## Abstract

**Background:**

Primary progressive aphasia (PPA) is a language‐based dementia linked with underlying Alzheimer’s disease (AD) or frontotemporal dementia. Clinicians often report difficulty differentiating between the logopenic (lv) and nonfluent/agrammatic (nfv) subtypes, as both variants present with disruptions to “fluency” yet for different underlying reasons. In English, acoustic and linguistic markers from connected speech samples have shown promise in machine learning (ML)‐based differentiation of nfv from lv. To our knowledge, this approach has not been evaluated in other languages nor in the context of bilingualism.

**Method:**

Twenty‐four Spanish‐Catalan bilingual patients (lv = 15, nfv = 9) were asked to describe a picture (WAB Picnic Scene) in both their dominant and non‐dominant language. From the participant’s recorded response, 10 acoustic features were derived with PRAAT and 15 linguistic features were derived with the Natural Language Processing (NLP) tools SpaCy and CLAN. A similarity score between the image and patient’s transcription was derived with the Vision‐Language model CLIP. The acoustic features, linguistic features, and CLIP scores, were separately fed into ML classification algorithms for differentiating nfv from lv in participants' dominant and non‐dominant samples.

**Result:**

The acoustic‐based classifiers achieved classification accuracy (F1 score) of 59% in the dominant and 86% in the non‐dominant language, respectively. The linguistic‐based classifiers achieved F1 scores of 73% in the dominant and 77% in the non‐dominant language, respectively. The CLIP‐based classifier achieved F1 scores of 82% in the dominant and 82% in the nondominant language, respectively. The acoustic and linguistic classifier performed 25% (p = 0.077) and 4% (p = 0.18) better given only non‐dominant samples compared to only dominant samples.

**Conclusion:**

Taking advantage of recent advances in multilingual NLP, we achieved promising and effective differentiation of nfv from lv for Spanish‐Catalan bilingual patients using a nearly automated pipeline. Interestingly, our acoustic and linguistic‐based classifiers performed better given responses from a patient’s non‐dominant language, and the acoustic feature set was more accurate in discriminating between nfv and lv compared to the linguistic model. Future directions include examining patterns in a larger sample size and comparison of performance on different types of connected speech tasks.